# MRM screening/biomarker discovery with linear ion trap MS: a library of human cancer-specific peptides

**DOI:** 10.1186/1471-2407-9-96

**Published:** 2009-03-27

**Authors:** Xu Yang, Iulia M Lazar

**Affiliations:** 1 Virginia Bioinformatics Institute, Virginia Polytechnic Institute and State University, Blacksburg, VA 24061, USA; 2 Department of Biological Sciences, Virginia Polytechnic Institute and State University, Blacksburg, VA 24061, USA

## Abstract

**Background:**

The discovery of novel protein biomarkers is essential in the clinical setting to enable early disease diagnosis and increase survivability rates. To facilitate differential expression analysis and biomarker discovery, a variety of tandem mass spectrometry (MS/MS)-based protein profiling techniques have been developed. For achieving sensitive detection and accurate quantitation, targeted MS screening approaches, such as multiple reaction monitoring (MRM), have been implemented.

**Methods:**

MCF-7 breast cancer protein cellular extracts were analyzed by 2D-strong cation exchange (SCX)/reversed phase liquid chromatography (RPLC) separations interfaced to linear ion trap MS detection. MS data were interpreted with the Sequest-based Bioworks software (Thermo Electron). In-house developed Perl-scripts were used to calculate the spectral counts and the representative fragment ions for each peptide.

**Results:**

In this work, we report on the generation of a library of 9,677 peptides (p < 0.001), representing ~1,572 proteins from human breast cancer cells, that can be used for MRM/MS-based biomarker screening studies. For each protein, the library provides the number and sequence of detectable peptides, the charge state, the spectral count, the molecular weight, the parameters that characterize the quality of the tandem mass spectrum (p-value, DeltaM, Xcorr, DeltaCn, Sp, no. of matching ***a***, ***b***, ***y ***ions in the spectrum), the retention time, and the top 10 most intense product ions that correspond to a given peptide. Only proteins identified by at least two spectral counts are listed. The experimental distribution of protein frequencies, as a function of molecular weight, closely matched the theoretical distribution of proteins in the human proteome, as provided in the SwissProt database. The amino acid sequence coverage of the identified proteins ranged from 0.04% to 98.3%. The highest-abundance proteins in the cellular extract had a molecular weight (MW)<50,000.

**Conclusion:**

Preliminary experiments have demonstrated that putative biomarkers, that are not detectable by conventional data dependent MS acquisition methods in complex un-fractionated samples, can be reliable identified with the information provided in this library. Based on the spectral count, the quality of a tandem mass spectrum and the m/z values for a parent peptide and its most abundant daughter ions, MRM conditions can be selected to enable the detection of target peptides and proteins.

## Background

The identification of novel protein biomarkers for early disease detection, risk assessment, treatment, prediction of therapeutic response or toxicity, will dramatically improve disease outcomes and survivability rates. The discovery process of protein biomarkers relies, essentially, on the detection and quantitation of protein differential expression patterns in diverse samples [1-4]. Recently, mass spectrometry has evolved into a powerful tool for the analysis of complex proteomic extracts, and various quantitative proteomic approaches (label-free/stable isotope labeling or absolute/relative) have been developed [5-15]. Large-scale quantitation is typically accomplished by comparing the sample of interest to a pre-defined reference sample of similar complexity. The classical data-dependent driven MS/MS profiling technique, in which an attempt is made to detect all components in a proteome, has provided limited reproducibility for quantitation purposes and limited capability for detecting low abundant proteins, such as the case of many biomarkers. At the cost of restricting the discovery potential, a targeted screening approach, i.e., multiple reaction monitoring, has been developed to enable the reliable detection and quantitation of representative peptides for selected proteins. While MRM is one of the most sensitive MS scanning modes for peptide identifications, it is best applicable to previously identified peptides with known MS/MS fragmentation pattern [16,17]. An MRM experiment is conducted by selecting representative peptides of a protein with known m/z values (precursor ions), fragmenting them through collision induced dissociation (CID), and monitoring only specific, pre-selected daughter fragments (product ions) that are characteristic to each precursor. The combination of a precursor-product m/z values is known as a 'transition,' and is highly specific for a given peptide amino acid sequence. As only a narrow mass range around the m/z of the daughter ion is monitored by MS, the method provides for a fast and sensitive detection of selected peptides. When combined with methods that rely on the use of stable isotope-labeled peptide standards, this approach can be successfully applied for the absolute and relative quantitation of low abundant components in complex samples. With this method, 47 high/intermediate-abundance proteins were quantified successfully in human plasma (<1 μg/mL level, coefficient of variation, CV = 2–22%) [18], and C-reactive protein [19], human growth hormone [20], and prostate-specific antigen [21] were measured in plasma or serum. Alternatively, MRM-based approaches have been used to identify the presence of phosphorylation on key cell cycle regulatory proteins [22], to quantify multisite phosphorylation [23], and to perform quantitative proteomic analysis of cellular signaling networks [24].

In most MS quantitative studies, the instrument of choice is a triple quadrupole mass spectrometer. Recently, a new type of MS instrument, i.e., the linear ion trap, has gained popularity among proteomics researchers. In a triple quadrupole instrument, CID is accomplished by accelerating the precursor ions in a dc/rf electrical field to induce fragmentation through successive collisions with background gas molecules (multi-step fragmentation). In an ion trap instrument, CID is accomplished by exciting the precursor ions at their resonant frequency. As the product ions have different masses than the precursor ion, they are not in resonance with the excitation frequency, and are not subjected to further ion fragmentation as in a triple quadrupole instrument (single-step fragmentation). Thus, the analysis of large peptides by MRM in linear ion-trap mass spectrometers can be performed with improved detection limits, due to the formation of fewer but more intense product ions in the ion trap vs. the triple quadrupole [25,26]. By using one-dimensional chromatographic separations and linear ion trap MS detection, the quantitation of 5 intermediate-abundance serum proteins by MRM, with good precision and accuracy, at ~1–30 μg/mL levels, was reported [27].

In order to perform MRM experiments, the m/z of a specific peptide precursor and its selected product ions must be known. Large-scale proteomic analyses on various mass spectrometry platforms have revealed that proteins are consistently identified by only a handful of possible tryptic peptides, and that frequently observed peptides are not necessarily generated from the most abundant proteins. The peptides that are preferentially observed for a protein are called "proteotypic" [28-30]. For example, Mallick *et al*. have classified a peptide as being proteotypic if it was observed in >50% of all identifications of a corresponding protein (based on data obtained from large yeast proteomic archives), and evaluated 494 numeric physicochemical property scales for amino acids (e.g., charge, secondary structure, hydrophobicity, etc.) to develop a computational tool that can predict the proteotypic propensity of a peptide [28]. In addition, machine-learning algorithms have been developed to generate information related to the peptide fragmentation pattern [31]. Nevertheless, such computational predictions are often mass spectrometry platform dependent. Tandem mass spectra of proteotypic peptides, most commonly generated on quadrupole or 3D ion trap instruments, have been collected so far in databases such as PeptideAtlas [32], GPM [33] and PRIDE [34]. Due to differences in the CID process, as discussed earlier, triple quadrupoles and ion traps often generate different peptide fragmentation patterns (i.e., different product ion species with different intensities), and to date, very few data generated by linear ion trap instruments have been made available through public repositories. In this work, we provide human breast cancer tandem mass spectrometry data generated on a linear ion trap instrument (LTQ/Thermo) that were collected into a library of 1,572 proteins matched by a list of 9,677 peptides. Among many parameters, the spectral count for each ion species, the best p-value, and the top 10 most intense daughter ions are provided to enable the selection of the most frequently identified peptides for MRM proteomic explorations. Validation of protein identifications, and relative/absolute protein quantitation for biomarker discovery or screening, are envisioned to be the most relevant applications that would benefit from the information provided in this table.

## Methods

### Cell culture and processing

MCF-7 cells were cultured in EMEM with 10 μg/mL bovine insulin and 10% FBS, in an incubator maintained at 37°C with 5% CO_2 _[35]. At 70% confluence the cells were harvested, rinsed three times with phosphate buffer saline (PBS), and lysed by rocking at 4°C (2 h) with a lysis solution prepared from 1 mL RIPA buffer (500 mM TrisHCl pH 7.4, 1.5 M NaCl, 10% NP-40, 2.5% deoxycholic acid, 10 mM EDTA), 100 μL protease inhibitor cocktail (104 mM AEBSF, 0.08 mM aprotinin, 2 mM leupeptin, 4 mM bestatin, 1.5 mM pepstatin A, 1.4 mM E-64), phosphatase inhibitors [100 μL NaF (~100 mM) and 50 μL Na_3_VO_4 _(~200 mM)], and 8.75 mL of ice cold water. After centrifugation at 13,000 rpm and 4°C (15 min), the supernatant was collected and its protein concentration was measured by using the Bradford assay performed at 595 nm with a SmartSpec Plus Spectrophotometer (Bio-Rad, Hercules, CA, USA). Protein digestion was performed by first treating the protein extract with urea (8 M) and DTT (4.5 mM) at 60°C (1 h) to denature the proteins and cleave the disulfide bonds, followed by 10× dilution with 50 mM NH_4_HCO_3 _and trypsinization at 37°C for 24 h (50:1 w/w protein:enzyme ratio). The protein digest was desalted with SPEC-PTC18 solid phase extraction pipette tips (Varian Inc., Lake Forest, CA, USA), concentrated to ~4 mg/mL, and stored at -80°C prior to further analysis.

### SCX-LC-MS/MS

SCX-LC-MS/MS analysis was performed with a micro liquid chromatography system (Agilent Technologies, Palo Alto, CA, USA) interfaced to an LTQ ion trap mass spectrometer (Thermo Electron Corp., San Jose, CA, USA). The protein digest was prefractionated into 16 sample sub-fractions using a Zorbax Bio SCX Series II column (0.8 mm i.d. × 5 cm) from Agilent Technologies. Solvent A was H_2_O/CH_3_CN (95:5 v/v) supplemented with 0.1% HCOOH, and solvent B was H_2_O/CH_3_CN (95:5 v/v) supplemented with 0.1% HCOOH and 500 mM NaCl. The eluent gradient ran from 0 to 100% B (50 min) at a flow rate of 20 μL/min. Each SCX sub-fraction was analyzed by RPLC-MS/MS. Reversed phase columns were prepared in-house from fused silica capillaries [100 μm i.d. × (8–12 cm) long] packed with 5 μm Zorbax SB-C18 particles (Agilent Technologies), and connected to 1 cm long (20 μm i.d. × 90 μm o.d.) nanospray emitters to enable electrospray ionization (ESI)-MS analysis. The ESI voltage was 2,000 V. Solvent A was H_2_O/CH_3_CN (95:5 v/v) and solvent B was H_2_O/CH_3_CN (20:80 v/v), both supplemented with 0.01% trifluoroacetic acid (TFA). The split flow rate through the HPLC column was ~160–180 nL/min. The eluent gradient was from 10 to 100% B, the gradient length being 80–215 min long. Three sets of SCX samples (48 sample sub-fractions) were subjected to LC-MS/MS analysis (total sample analyzed estimated to be ~15–42 μg per set). Alternatively, ten MCF-7 protein extract digests were cleaned up with SPEC-PTC18 and SPEC-PTSCX cartridges, and analyzed directly by LC-MS/MS without prior SCX fractionation. As the sample amount subjected to analysis was different for every SCX set, the length of the μLC columns and the LC gradients varied from one set of analyses to another.

### Tandem MS data analysis

Data dependent MS analysis was performed by acquiring one MS scan (5 microscans averaged) followed by one zoom scan (5 microscans averaged) and one MS^2 ^on the top 5 most intense peaks. The zoom scan width was ± 5 m/z, and the dynamic exclusion was enabled at repeat count 1, exclusion list size 200, exclusion duration 60 s, and exclusion mass width ± 1.5 m/z. Collision induced dissociation was performed by setting the ion isolation width at 3 m/z, normalized collision energy at 35%, activation Q at 0.25, and activation time at 30 ms. The combined results of 48 SCX-LC-MS/MS and 10 LC-MS/MS runs were used to perform protein database searching. Protein identification was performed with the Bioworks 3.3 software (Thermo Electron Corp, San Jose, CA, USA) by using a minimally redundant database downloaded from SwissProt (37,678 entries) on January 2008. The database search parameters were chosen as follows: only fully tryptic fragments were considered in the analysis, the number of allowed missed cleavage sites was 2, the peptide tolerance was 2 amu, and the fragment ion tolerance was 1 amu. Chemical and/or posttranslational modifications were not allowed. The capability to match one peptide sequence to multiple protein references within the database was not enabled. MRM data acquisition was performed using the same CID parameter settings as for data dependent analysis, and included the development of LC-MS/MS runs with 1–6 segments (20–240 min long) and 6–9 scan events/segment. Specific conditions for each transition are discussed in the following sections of the manuscript.

### Reagents

MCF-7 cells and cell culturing reagents [Eagle's Minimum Essential Medium (EMEM), fetal bovine serum (FBS), insulin, trypsin/EDTA for cell detachment] were purchased from ATCC (Manassas, VA USA). RIPA lysis buffer was obtained from Upstate (Lake Placid, NY, USA). Protease inhibitor cocktail, phosphatase inhibitors, NaCl, TFA, HCOOH, TrisHCL, urea and DTT were purchased from Sigma (St. Louis, MO, USA). Sequencing grade modified trypsin was from Promega Corp. (Madison, WI, USA). NH_4_HCO_3 _was purchased from Aldrich (Milwaukee, WI, USA). HPLC grade acetonitrile was purchased from Fisher Scientific (Fair Lawn, NJ, USA), and deionized water (18 MΩ -cm) was generated using a MilliQ ultrapure water system (Millipore, Bedford, MA, USA).

## Results and discussion

### Library construction and content

Large scale proteomic studies on MCF-7 and/or other breast cancer cell lines have resulted in the combined identification of ~1,000–4,000 proteins by using 2D-gel electrophoresis or shotgun analysis protocols (false positive rates of <5%) [35-38]. In this work, a protein/peptide library was generated from 58 LC-MS/MS data dependent analyses (see Additional file 1: **Appendix 1**). Tandem MS data were filtered at the peptide level with the Xcorr vs. charge state filter set at Xcorr = 1.5 for z = 1, Xcorr = 2.0 for z = 2, and Xcorr = 3.0 for z ≥ 3, respectively, and at the protein level by considering only proteins with ≥ 2 spectral counts. A total of 2,286 proteins (p < 0.001) were identified. The library comprises 1,572 proteins (all with ≥ 2 spectral counts) matched by 9,677 peptides (all with p < 0.001, p being the probability of a random match as calculated by the Bioworks software). By using such MS data filtering parameters and by selecting only proteins and peptides with p < 0.001, the rate of false positive identifications [39] when searching the data against a forward/reversed human protein database was ~1.5% and ~4.5% at the peptide and protein levels, respectively. At the protein level, the library provides the p-value, the score, the sequence coverage, the molecular weight, and the number of total and unique peptides observed for each protein. The total number of observed peptides (or the peptide hits) represents the spectral count. In addition, based on the protein sequences provided in the SwissProt database, we calculated the theoretically observable peptides, i.e., the tryptic peptides with maximum 2 missed cleavages (we note that the raw data were searched against the human database by allowing for such peptides in the search). The ratio of the unique observed to observable peptides is an indicative of the protein abundance, and was previously coined as the protein abundance index-PAI [40,41]. At the peptide level, the library provides the amino acid sequence of each peptide, the charge state, the spectral count of each peptide at each identifiable charge state, the protonated mass (MH^+^), the parameters that characterize the quality of a tandem mass spectrum [DeltaM, p-value, Xcorr, DeltaCn, Sp, the # of matching ions (***b***, ***y ***and ***a***) in the tandem mass spectrum], the retention time of the peptide, the length of the LC gradient (10 to 100% B), and 10 product ions from each tandem mass spectrum for MRM analysis. As every peptide sequence generated several tandem mass spectra, the data from **Appendix 1 **(see Additional file 1) correspond to the spectra with the best (i.e., the lowest) p-value. Four in-house developed Perl-scripts were used to generate the library. The first Perl-script was used to calculate the spectral count (from all 58 LC-MS/MS experiments) for each unique amino acid sequence peptide at a given charge state, and to select the best tandem mass spectrum for this peptide (i.e., the mass spectrum with the lowest p-value). A second Perl-script was used to select representative ions for MRM analysis. The strategy involved the extraction of the top 10 most intense daughter ions from the DTA file associated with the best tandem mass spectrum of a peptide. Ions in the vicinity of the parent (m/z_parent _± 60) were excluded to avoid the selection of adducts or neutral loss ions. In addition, ions in the immediate vicinity of a fragment (m/z_fragment _± 3) that was already selected for MRM were excluded, as well, to avoid duplication by the selection of isotopic peaks. The third Perl-script was used to calculate the observable peptides for each protein. The algorithm involved performing in-silico tryptic digestion for each protein in the SwissProt database, and counting the number of peptides with mass ranging from 500 to 4,000 Da and with 0, 1 or 2 missed cleavages. The fourth Perl-script was used to extract the LC retention time of each peptide from the Sequest result files.

### Data evaluation

**Figure 1:**
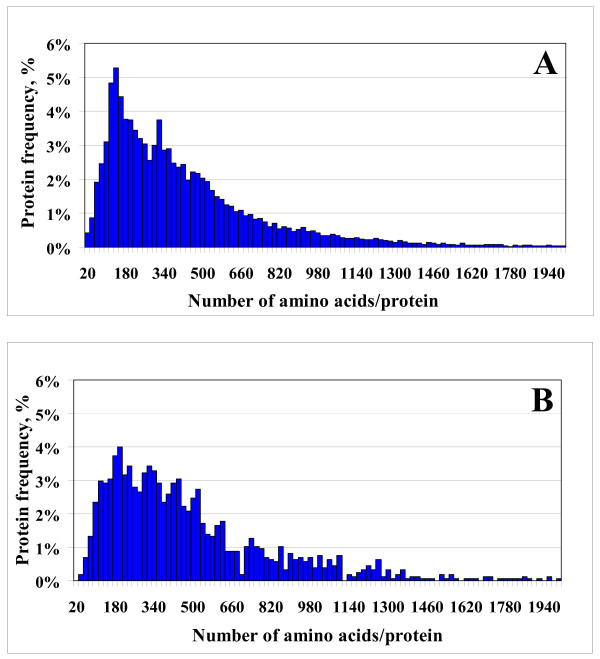
**Proteomic maps**. (**A**) Theoretical distribution of the human proteins according to the SwissProt database (~25,000 genes); (**B**) Experimental distribution of the 1,572 proteins from the MCF-7 library (all proteins were identified with p < 0.001 and ≥ 2 spectral counts).

**Figure 2:**
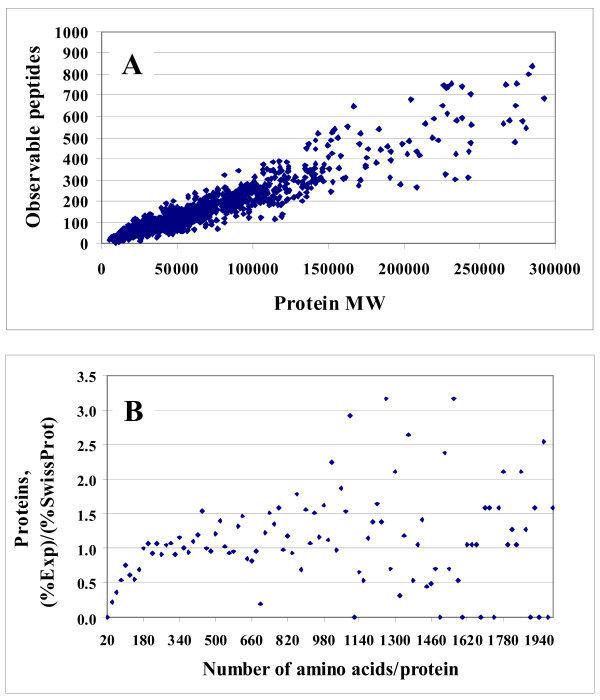
**Charts that illustrate the impact of protein size on likelihood of detection**. (**A**) Chart illustrating the distribution of observable (theoretical) tryptic peptides as a function of protein MW, for the set of 1,572 proteins; (**B**) Chart illustrating the ratio of the experimental percentage of identified proteins to the theoretical percentage of proteins *vs*. the number of amino acids in a protein. The experimental protein percentages were calculated relative to the total number of identified proteins, and the theoretical percentages were calculated relative to the total number of proteins in the SwissProt database.

To obtain a qualitative view of how well this protein pool represented the human proteome, a chart reflecting the experimental frequency of the 1,572 identified proteins as a function of MW (that ranged from ~5,000 to ~1,000,000 Da) was constructed, and compared to a similar chart reflecting the theoretical protein distribution downloaded from the SwissProt/Expasy website http://www.expasy.ch (see Figure 1). The MW was expressed in terms of number of amino acids per protein, by assigning to each amino acid the molecular weight of averagine (i.e., MW = 111.12) [42]. The experimental and theoretical distributions were fairly similar, illustrating that our dataset comprised a representative set of proteins, and that our experimental protocol performed well in sampling the human proteome. A small bias towards proteins with a larger number of amino acids, was, however, observed. It was noticed that proteins with a sequence shorter than 200 amino acids (MW~22,200) were less frequently encountered. The theoretical and experimental protein distributions peaked at proteins containing 140–160 and 180–200 amino acids, respectively. Similar results were obtained if all proteins with p < 0.001, not just the ones with two spectral counts, were considered in the comparison. Assuming that there was no bias introduced by losing peptides belonging to small MW proteins during sample processing (e.g., by protein digestion, recovery of peptides from clean-up cartridges, etc.), we attributed this bias to a lower sampling rate during MS data dependent analysis, as a result of a smaller number of matching tryptic peptides that can be generated from low MW proteins. We would expect that large MW proteins will generate a larger number of peptides, increasing, thus, the likelihood of detection during a data dependent analysis process. For this data set (1,572 proteins), the increase in observable (theoretical) tryptic peptides with the protein MW is shown in Figure 2A, and the ratio of experimental percentage of identified proteins to the theoretical percentage (according to the SwissProt chart) *vs*. the number of amino acids in a protein, is shown in Figure 2B. The range of 20–1,940 amino acids/protein corresponds to a range of MW of 2,222<MW<215,573. Low MW proteins are clearly under-sampled in our extract, and the number of available peptides/protein for MS detection could provide at least a partial explanation for a more successful mapping, in terms of numbers, of high MW proteins. However, the dynamics of protein turnover is an additional factor that may affect the success of MS detection in complex cellular extracts. Effective sampling of a proteome, in a relevant biological context, will have to take into account correlations between protein function, protein half-live (that can vary from minutes to hours or days), and eventually protein MW.

**Figure 3:**
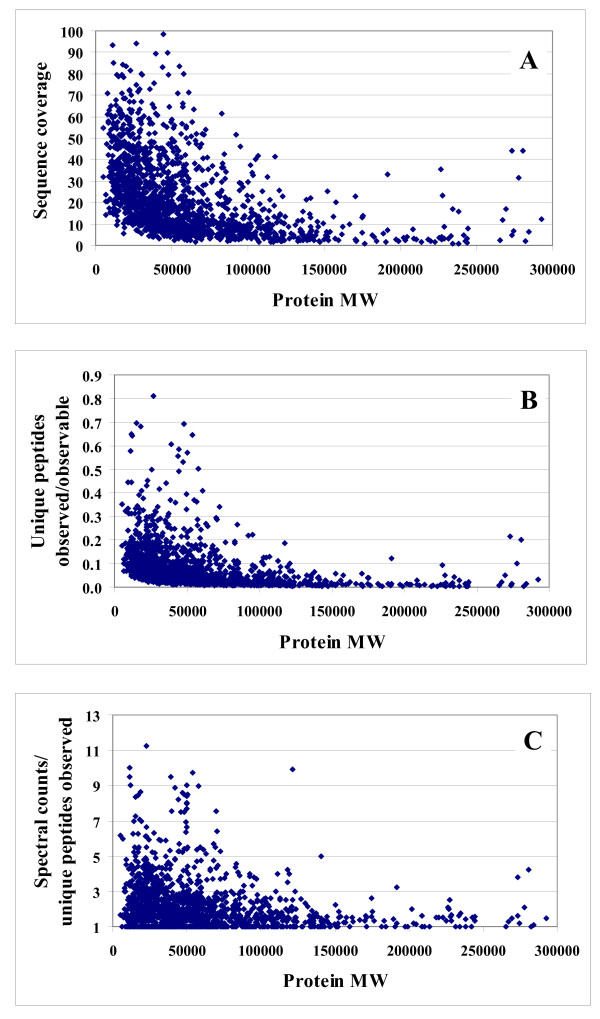
**Charts that illustrate protein abundance as a function of protein MW, for the set 1,572 proteins**. (**A**) Chart illustrating protein abundance as a function of MW in terms of experimental protein sequence coverage; (**B**) Chart illustrating protein abundance as a function of MW in terms of observed/observable unique peptides; (**C**) Chart illustrating protein abundance as a function of MW in terms of spectral counts/observed unique peptides.

Protein detectability is not only dependent on the number of observable peptides/protein, but also on the protein abundance and the proteotypic propensity of the matching peptides, and can be assessed in terms of sequence coverage. For this data set, we note that while the overall sequence coverage of the identified proteins was fairly broad (i.e., 0.04%–98.3%), the low MW proteins were clearly indentified with a higher sequence coverage despite the smaller number of unique peptides/protein (Figure 3A). The observed number of unique or total peptide hits (spectral counts), while dependent on the protein MW, is also a strong indicative of the protein abundance and of the peptide propensity for MS identification. This quantitative relationship is represented in Figure 3B for unique peptides, and in Figure 3C for total spectral counts. To eliminate the bias introduced by high MW proteins generating more peptides, Figure 3B displays the ratio of the experimentally observed unique peptides to the theoretically observable peptides as a function of protein MW. Proteins with MW<50,000 were found to be more abundant in the cellular extract, the most abundant proteins peaking out at MW~20,000–30,000. We must note, however, that many experimental factors can affect the interpretation of results. For example, the extraction, denaturation and tryptic digestion of proteins could correlate negatively with the MW of proteins, resulting, thus, in a lower number of observed peptides/protein. We should also note that peptides with propensity for identification will generate progressively increased spectral counts at higher abundance levels, as they elute as broader chromatographic peaks during LC-MS/MS analysis. The chart that is displayed in Figure 3C eliminates the impact of peptide propensity for detection by providing the number of spectral counts/number of experimentally detected unique peptides as a function of protein MW, and strengthens the conclusion that proteins with MW<50,000 were, overall, more abundant (assuming that the MW of the originating proteins introduced no consistent bias in the proteotypic behavior of peptides). Further work will be, however, necessary to evaluate the impact of protein size, hydrophobic properties and packing on the effectiveness with which large MW proteins are processed and detected experimentally, to enable more general conclusions regarding the abundance of proteins in whole cellular extracts.

### MRM analysis

The information provided in the protein/peptide library can be effectively used to perform MRM experiments. The spectral count of each peptide, at the detectable charge state, reflects its propensity for identification (we note that not all peptides with high spectral count are necessarily proteotypic according to the definition provided in reference 28, i.e., that are detectable in >50% of the trials that identified the corresponding protein). The p-value and the other SEQUEST scores reflect the quality of the tandem mass spectrum that led to the identification of the peptide. Up to ten MRM transitions can be set up for each parent ion. By displaying only peptides with p < 0.001 [i.e., -10log(p)>30], it was ensured that the ions selected by the Perl script were mostly ***a***, ***b***, ***y***, **H**_2_**O**/**NH**_3_-neutral loss or multiple loss ions, but not noise or other contaminants. We note, however, that the experimental product ions were generated by enabling the database search with a fragment ion tolerance of 1 amu, thus contaminant product ions within this mass window are possible. Quick manual corroboration with software packages such as Protein Prospector http://prospector.ucsf.edu can confirm the validity of the product ions in the library, and help eliminate contaminant ions that do not belong to the considered peptide. Generally, the lower it is the p-value of a peptide [i.e., the higher the -10log(p)], the less likely it is the presence of extraneous fragment ions in the list.

**Figure 4:**
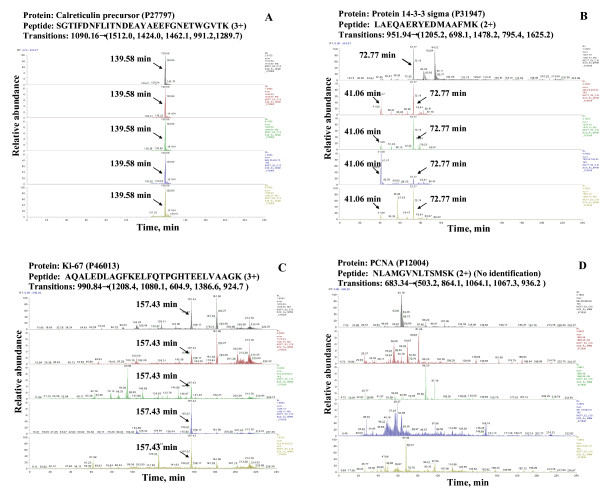
**Extracted ion chromatograms illustrating five MRM transitions/peptide for the identification of putative cancer biomarkers in whole cellular extracts**. Conditions: MCF-7 whole cellular extracts were digested with trypsin, cleaned-up with SPEC-PTSCX and SPEC-PTC18 cartridges, and analyzed by a ~4 h long LC-MS/MS gradient. The top-down order of EICs reflects the order of the five transitions shown in Table 1.

The applicability of this peptide library for the identification of putative biomarkers in proteomic samples is demonstrated with a few examples that involved the analysis of un-fractionated MCF-7 protein extracts. Whole cellular extracts represent a good testing system for demonstrating the effectiveness of MRM analysis, as due to complexity, the extracts do not facilitate the detection of low abundance components. When using a data dependent acquisition process, such extracts typically enabled the identification of only ~400–600 proteins with p < 0.001 (~200–300 proteins with 2 spectral counts) per LC-MS/MS run, i.e., ~5 times less than the SCX prefractionated samples that enabled the identification of ~2,000 proteins [35]. The following scenarios were encountered during data dependent analysis of a whole cellular extract: (1) the protein and all matching peptides from the library were identifiable; (2) the protein was identifiable by some, but not all matching peptides from the library; and (3) the protein was not identifiable by any of the peptides listed in the library. The detection of a set of seven putative biomarker proteins, as previously reported in the literature [43-45], was facilitated by enabling MRM transitions for the corresponding peptides that are shown in Table 1. The proteins and the peptides that were not detectable in the whole extract by data dependent analysis are marked with "**no ID**." Peptides from the library with the largest number of spectral counts and best SEQUEST scores (most importantly with the lowest p-values) were selected for MRM analysis. The product ions that were monitored for these peptides were the first five most intense. Representative results of extracted ion chromatograms (EIC) for these transitions are summarized in Figure 4. As the LTQ is a relatively low mass accuracy/resolution instrument, the mass window that was monitored around a product ion in the EIC was fairly broad (m/z = ± 1.5), enabling, thus, contaminant fragments to interfere with the MRM analysis. However, the ability to detect all transitions at the retention time of the parent peptide can greatly increase the specificity of detection, as contaminants with the same precursor m/z, same fragment(s) m/z, and same retention time, are highly unlikely.

**Table 1:** MRM transition chart for the identification of putative protein biomarkers.

Protein/Peptides	z	-10lg(p)	Spectral count	MRM range (min)	Transitions (MH+ → product ions)
**O43399|TPD54_Human Tumor Protein D54**					

GLLSDSMTDVPVDTGVAAR	2	91	2	40-90	952.47→(885.3, 1019.2, 1200.3, 1099.2, 574.3)

VVGDRENGSDNLPSSAGSGDKPLSDPAPF (**no ID**)	3	136	15	0-240	962.45→(1312.0, 1228.1, 1127.0, 1256.3, 815.9)

LGLSTLGELKQNLSR (**no ID**)	2	114	5	0-240	814.97→(1044.3, 617.3, 745.3, 673.4, 1157.4)

TPAVEGLTEAEEEELRAELTKVEEEIVTLR	3	300	8	145-170	1128.58→(1508.1, 1443.7, 1358.6, 1063.0, 1244.1)

**P31947|1433S_Human 14-3-3 protein sigma**					

LAEQAERYEDMAAFMK	2	112	4	40-90	951.94→(1205.2, 698.1, 1478.2, 795.4, 1625.2)

VLSSIEQKSNEEGSEEKGPEVR (**no ID**)	3	133	9	0-240	811.06→(740.6, 1110.0, 966.5, 1080.0, 557.2)

**P27797|CALR_Human Calreticulin precursor**					

SGTIFDNFLITNDEAYAEEFGNETWGVTK	3	300	25	125-145	1090.16→(1512.0, 1424.0, 1462.1, 991.2,1289.7)

FYGDEEKDKGLQTSQDAR	2	141	13	0-40	1043.98→(1841.9, 803.6, 975.5, 1112.6, 1104.6)

IDNSQVESGSLEDDWDFLPPKK	3	85	14	0-240	840.40→(981.9, 1146.5, 932.8, 1089.3, 1204.0)

**P08195|4F2_Human 4F2 cell-surface antigen heavy chain**					

ADLLLSTQPGREEGSPLELER	3	84	5	40-90	770.73→(706.2, 893.3, 900.6, 842.4, 1004.3)

IKVAEDEAEAAAAAK	2	94	5	0-240	743.89→(1245.2, 1146.1, 946.1, 831.1, 1075.1)

**P07339|CATD_Human Cathepsin D precursor**					

ISVNNVLPVFDNLMQQK	2	119	22	90-125	980.02→(1219.3, 1332.3, 880.5, 610.1, 724.2)

LVDQNIFSFYLSRDPDAQPGGELMLGGTDSK	3	300	23	0-240	1124.21→(1261.3, 1344.6, 1055.2, 1016.1, 1579.6)

**P46013|Ki-67 antigen (no ID)**					

AQALEDLAGFKELFQTPGHTEELVAAGK (**no ID**)	3	71	1	0-240	990.84→(1208.4, 1080.1, 604.9, 1386.6, 924.7)

SGGSGHAVAEPASPEQELDQNKGK (**no ID**)	3	44	1	0-240	798.38→(956.0,870.9, 643,2, 921.0, 653.1)

**P12004|PCNA_HUMAN Proliferating cell nuclear antigen (no ID)**					

ATPLSSTVTLSMSADVPLVVEYK (**no ID**)	2	106	4	0-240	1204.63→(847.2, 1561.2, 1061.2, 1437.3, 538.1)

FSASGELGNGNIK (**no ID**)	2	98	7	0-240	647.32→(716.1, 744.2, 546.1, 902.2, 989.2)

LSQTSNVDKEEEAVTIEMNEPVQLTFALR (**no ID**)	3	101	5	0-240	1097.88→(1559.1, 1551.3, 1393.6, 1467.1, 937.4)

YYLAPKIEDEEGS (**no ID**)	2	112	8	0-240	757.35→(1003.5, **676.4**, 511.2, 1074.5, 1187.6)

DLSHIGDAVVISCAK (**no ID**)	2	113	4	0-240	764.39→(962.2, **566.1**, 606.9, 650.4, 1075.2)

NLAMGVNLTSMSK (**no ID**)	2	35	5	0-240	683.34→(**503.2, 864.1, 1064.1, 1067.3, 936.2**)

Peptides or proteins marked with "**no ID**" were not detectable in the whole protein extract by data dependent MS analysis. Transitions that did not result in peptide identification in the EIC are marked in **bold**.

When a protein was detectable in the whole cell extract by data dependent analysis, very strong product ion peaks were observable in the EIC of each transition that was enabled for a peptide (see Figure 4A, peptide at 139.58 min). In Figure 4A, each transition was enabled for 20 min. Nevertheless, such transitions can be enabled for a much shorter time, when the retention time (t_r_) of a peptide is well controlled, or for longer times, or even for the entire length of the experiment, when the t_r _is not known. Given that the LC-MS/MS analyses that contributed with data to this library were conducted for different lengths of time, the peptide t_r_(s) in Figure 4 do not correspond to the t_r_(s) from the library. To enable a rough prediction of a peptide t_r_, the length of each LC gradient (10–100% B) is also provided. We note that (a) both retention time and gradient length include a ~20 min dead-time corresponding to the elution of non-retained components from the LC column, and (b) the gradient was not linear, as 80–90% of the gradient length was dedicated to increase the % B from 10 to 45%. Later experiments in our lab have confirmed that *retention time*/*gradient length *estimates could be obtained within ± 10–25% of the values provided in the library.

The presence of contaminant peptide species with close m/z to the peptide of interest, and with several overlapping transitions, was observed in our MRM studies (see Figure 4B, monitored peptide at 72.77 min, contaminant peptide at 41.06 min). Interference from such peptides can be eliminated by narrowing the m/z window that is used for the generation of the EIC, or by narrowing the time window that is used for monitoring the MRM transition (when the elution time of the peptide is known). For example, if the MRM transitions would have been enabled only from 70 min to 75 min, the contaminant peptide at 41.06 min would have not interfered with the analysis.

In cases when the protein was not detectable in the whole extract by data dependent analysis (such as the case of Ki-67 and PCNA proteins), many of the matching peptides were still detectable by most, if not all, MRM transitions (see Figure 4C, peptide at 157.43 min). Figure 4C presents the case of a Ki-67 peptide for which MRM transitions were enabled for the entire length of the LC-MS/MS experiment. Even in the presence of contaminating transitions, the identification of the peptide could be confirmed by the detection of all predicted transitions at the expected t_r_. For other peptides, certain transitions were not observable in the EIC. Such transitions are shown in bold in Table 1. Missed transitions were especially observable in the case of library peptides identified by only one spectral count and p-values that were just above the threshold set for elimination from the list. For example, in the case of PCNA, the identification of peptide NLAMGVNLTSMSK was not conclusive based on the transitions that were provided in the library (Figure 4D), i.e., consistent transitions at the predicted peptide t_r _were not observable. Cross validation with Protein Prospector revealed that the first three transitions were probably not even correct for this peptide. The protein was, however, identifiable by MRM transitions enabled for other matching peptides.

## Conclusion

In summary, through this work, we make available for public use tandem MS information generated for a list of 1,572 proteins from MCF-7 human breast cancer cells. Unlike publically available empirical databases, our library provides a large set of proteins and peptides that can be identified in human cancerous cells under a consistent set of experimental conditions. As the data were generated with a linear ion trap mass spectrometer, the library strategically complements existing information generated with ESI-quadrupole (Q), ESI-Q-time-of-flight (TOF), ESI-ion cyclotron resonance (ICR) or matrix assisted laser desorption ionization (MALDI)-TOF instruments. Moreover, the availability of spectral count data provides information related to the abundance and proteotypic propensity of peptides, at given charge states, in the context of complex cellular extracts. The library enables the development of MRM-MS protocols for the identification of possibly hundreds of target proteins with particular relevance to biomarker screening and discovery applications. Key for the identification of a set of protein biomarkers in a complex un-fractionated cellular extract will be the development of MRM strategies that involve the selection of several peptides/protein (possibly with the highest spectral count and best SEQUEST scores) and of multiple transitions/peptide. Custom-prepared isotopically labeled versions of selected peptides could be further used for performing quantitation studies.

## Abbreviations

CID: collision induced dissociation; CV: coefficient of variation; DeltaCn: degree by which the lower ranked peptide scores differ from the correlation score of the best match; DeltaM: difference between the theoretical and experimental mass of a peptide; EIC: extracted ion chromatogram; ESI: electrospray ionization; ICR: ion cyclotron resonance; LTQ: linear trap quadrupole; MALDI: matrix assisted laser induced dissociation; MRM: multiple reaction monitoring; MS: mass spectrometry; MW: molecular weight; Q: quadrupole; RPLC: reversed phase liquid chromatography; Sp: preliminary score; SCX: strong cation exchange chromatography; TOF: time of flight; Xcorr: cross correlation score between virtual and experimental spectrum.

## Competing interests

The authors declare that they have no competing interests.

## Authors' contributions

XY developed the protein/peptide library, performed the MRM experiments, wrote the Perl scripts, and drafted the preliminary version of the manuscript. IML conceived the study, coordinated the work, evaluated the overall results, and prepared the final version of the manuscript. All authors have read and approved the final manuscript.

## Pre-publication history

The pre-publication history for this paper can be accessed here:

http://www.biomedcentral.com/1471-2407/9/96/prepub

## Supplementary Material

Additional file 1 **Protein/peptide library for MRM-MS-based detection of target proteins in human cancer cellular extracts.** The library provides tandem MS data for 9,677 peptides (p < 0.001) representing ~1,572 proteins from human breast cancer cells. Click here for file
